# Posttransplant Hemophagocytic Lymphohistiocytosis Driven by Myeloid Cytokines and Vicious Cycles of T-Cell and Macrophage Activation in Humanized Mice

**DOI:** 10.3389/fimmu.2019.00186

**Published:** 2019-02-13

**Authors:** Satoshi Yoshihara, Yuying Li, Jinxing Xia, Nichole Danzl, Megan Sykes, Yong-Guang Yang

**Affiliations:** ^1^Columbia Center for Translational Immunology, Department of Medicine, Vagelos College of Physicians and Surgeons, Columbia University, New York, NY, United States; ^2^Institute of Translational Medicine, First Hospital of Jilin University, Changchun, China

**Keywords:** allogeneic SCT, hypercytokinemia, inflammation, immune activation, hemophagocytic lymphohistiocytosis (HLH), graft-vs.-host disease, posttransplant complication

## Abstract

Hemophagocytic lymphohistiocytosis (HLH) has recently been increasingly reported as an important complication after stem cell transplantation, in line with the increase in the number of HLA-mismatched transplantation. Although previous clinical studies have shown an elevation of inflammatory cytokines in patients with HLH after hematopoietic stem cell transplantation, as well as those after viral infection or autoimmune disease, the disease pathogenesis remains poorly understood. Here we explored this issue in humanized mice with functional human lymphohematopoietic systems, which were constructed by transplantation of human CD34^+^ cells alone, or along with human fetal thymus into NOD/SCID/γc^−/−^ (NSG) or NSG mice carrying human SCF/GM-CSF/IL-3 transgenes (SGM3). In comparison with humanized NSG (huNSG) mice, huSGM3 mice had higher human myeloid reconstitution and aggressive expansion of human CD4^+^ memory T cells, particularly in the absence of human thymus. Although all huNSG mice appeared healthy throughout the observation period of over 20 weeks, huSGM3 mice developed fatal disease characterized by severe human T cell and macrophage infiltrations to systemic organs. HuSGM3 mice also showed severe anemia and thrombocytopenia with hypoplastic bone marrow, but increased reticulocyte counts in blood. In addition, huSGM3 mice showed a significant elevation in human inflammatory cytokines including IL-6, IL-18, IFN-α, and TNF-γ, faithfully reproducing HLH in clinical situations. Our study suggests that posttransplant HLH is triggered by alloresponses (or xenoresponses in our model), driven by myeloid cytokines, and exacerbated by vicious cycles of T-cell and macrophage activation.

## Introduction

Hemophagocytic lymphohistiocytosis (HLH), also known as hemophagocytic syndrome (HPS) or macrophage activation syndrome (MAS), is a disease triggered by hypercytokinemia (1). Although the most frequent trigger for HLH is viral infection, other immune activated conditions including autoimmune diseases and malignant lymphoma also serve as a trigger. Allogeneic transplantation may also induce inflammatory cytokine production as a consequence of tissue damages following preconditioning regimen and allogeneic immune responses. Indeed, HLH after allogeneic hematopoietic stem cell transplantation (allo-HSCT) has been increasingly reported (2–4). As well known, graft-vs.-host disease (GVHD) is also triggered by cytokine storm (5). While mechanisms of GVHD have been extensively examined, the pathogenesis of HLH remains poorly understood.

To explore this issue, we utilized humanized (hu) mice with functional human hematopoietic and lymphoid systems. Previous studies have shown that hu-mouse models serve as a highly useful tool to study human hematopoiesis, immune function, and various diseases (6). However, human myeloid reconstitution remains relatively poor in these hu-mice, presumably due to the lack of or insufficient cross-reactivity between mouse and human cytokines (7). To solve this problem, various strategies have been examined to express human myeloid cytokines in humanized mice (8–10). Besides the expression of human cytokines in the immunodeficient mice, there are considerable variations in the protocols used for the generation of hu-mice with a human immune system. The variations arise from human stem cell source (fetal, cord blood, or adult), age of mice (neonatal or adult), and the use of human thymus graft (11). We have previously shown that NSG mice receiving fetal thymic grafts and partially-matched allogeneic adult CD34^+^ cells show robust T-cell reconstitution, providing a new model for individualized analysis of human immune responses (12).

In this study we generated humanized mice by transplantation of human CD34^+^ cells either alone or combined with fetal thymic tissue into adult NSG or transgenic NSG mice expressing human stem cell factor (SCF), GM-CSF and IL-3 (referred to as SGM3 mice). We show that humanized SGM3 (huSGM3), but not huNSG, mice developed lethal HLH, regardless of whether or not human thymus was transplanted. Moreover, huSGM3 mice showed aberrant expansion of human T cells developing in the native mouse thymus, presumably reflecting poor negative selection of human thymocytes in the mouse thymus.

## Methods

### Animals and Human Tissues

NOD.Cg-Prkdc^scid^ Il2rg^tm1Wjl^/SzJ (NOD/SCID/γc^−/−^ or NSG) and NOD.Cg-Prkdc^scid^ Il2rg^tm1Wjl^ Tg(CMV-IL3,CSF2,KITLG)1Eav/MloySzJ (NSG-SGM3 or SGM3) mice were purchased from The Jackson Laboratory and were housed in a specific pathogen-free microisolator environment. Human fetal liver and thymus tissues of gestational age of 17–21 weeks were obtained from Advanced Bioscience Resource. Human CD34^+^ fetal liver cells (FLCs), isolated by a magnetic-activated cell sorter separation system using anti-CD34 microbeads (Miltenyi Biotec), and thymus tissues were cryopreserved in liquid nitrogen until use. Protocols involving the use of human tissues and animals were approved by the Institutional Review Board and the Institutional Animal Care and Use Committee of Columbia University (New York, NY), and all of the experiments were performed in accordance with the protocols.

### Humanized Mouse Preparation

Female NSG or SGM3 mice at the age of 10–11 weeks were conditioned with sublethal (1.2 Gy) total body irradiation using a RS2000 X-ray irradiator (Rad Source Technologies) and received human CD34^+^ FLCs (7 × 10^4^/mouse, intravenously) alone, or along with a human fetal thymic tissue fragment measuring approximately 1 mm^3^ (under the recipient kidney capsule) from the same fetal donor, as previously described (13–15). Humanized mice were monitored daily, body weight was checked weekly, and peripheral blood was collected from the retro-orbital sinus every 2–3 weeks starting 4 weeks after transplantation. RBC lysis using BD Pharm Lyse (BD Biosciences) was performed for leukocyte chimerism analysis, mononuclear cell purification by density gradient centrifugation (400 × *g* for 30 min at room temperature) with Histopaque 1077 (Sigma-Aldrich) was performed for human lymphocyte analysis, and whole blood was used for RBC chimerism analysis. Humanized mice were sacrificed when they became moribund and complete necropsy was performed.

### Isolation of Leukocytes From Organs in the Sacrificed Humanized Mice

Liver, spleen, lungs, and lymph nodes were minced and digested by Liberase TM (Roche) for 15 min at 37°C. Digested liver and lung cells were purified for mononuclear cells by density gradient centrifugation (400 × *g* for 30 min at room temperature) with Histopaque 1077 (Sigma-Aldrich). Digested spleen cells received RBC lysis by ACK lysing buffer (Lonza). Human thymus graft and mouse thymus were strained with a 40 μm nylon cell strainer (Falcon) to obtain a single cell suspension. The bone marrow (BM) cells, which were obtained from tibia and femur, received RBC lysis. Number of the cells were counted using a hemocytometer.

### Flow Cytometry

Flow cytometry was performed with LSR II (BD Biosciences) using various combinations of the following mAbs: anti-human CD45 (2D1), CD19 (HIB 19), CD3 (UCHT1), CD4 (RPA-T4), CD8 (SK1), CD33 (WM53), CCR7 (G043H7), CD45RA (HI100), CD31 (WM59), CD127 (A019D5), CD25 (M-A251), CD235a (HI264); anti-mouse CD45 (30-F11), and TER119 (TER-119); and isotype control mAbs (purchased from BD Biosciences PharMingen or Biolegend). Intracellular FoxP3 staining was performed with FoxP3 Staining Kit (Biolegend) according to the manufacturer's instructions.

### Cytologic and Histologic Analysis and Immunohistochemical Staining

Leukocytes isolated from organs underwent cytospin and Wright-Giemsa staining by conventional methods. Tissue samples underwent H&E staining and Prussian blue staining by conventional methods. Immunohistochemical staining was performed using rabbit anti-human CD3 antibody (SP7, Thermo Scientific) and mouse anti-human CD68 antibody (KP1, DAKO) as primary antibodies and appropriate secondary antibodies were used for detection.

### Quantification of WBC, Hemoglobin, Platelets, and Reticulocytes

Quantification of WBC, hemoglobin, platelets, and reticulocytes was performed using VetHemaChemRX (Oxford Science).

### Quantification of Cytokines in Plasma

Quantification of cytokines in cryopreserved plasma was performed by Luminex multiplex assay using ProcartaPlex® Multiplex Immunoassay Panels according to the manufacturer's instructions (eBioscience).

### Statistical Analyses

Statistical analysis was conducted using the Student's *t*-test, one-way ANOVA with Bonferroni's *post-hoc* multiple comparison test, two-way ANOVA, or log-rank test. A *P*-value of less than or equal to 0.05 was considered significant in all analyses herein.

## Results

### HuSGM, but Not HuNSG, Mice Develop Fetal Disease

**Figure 1:**
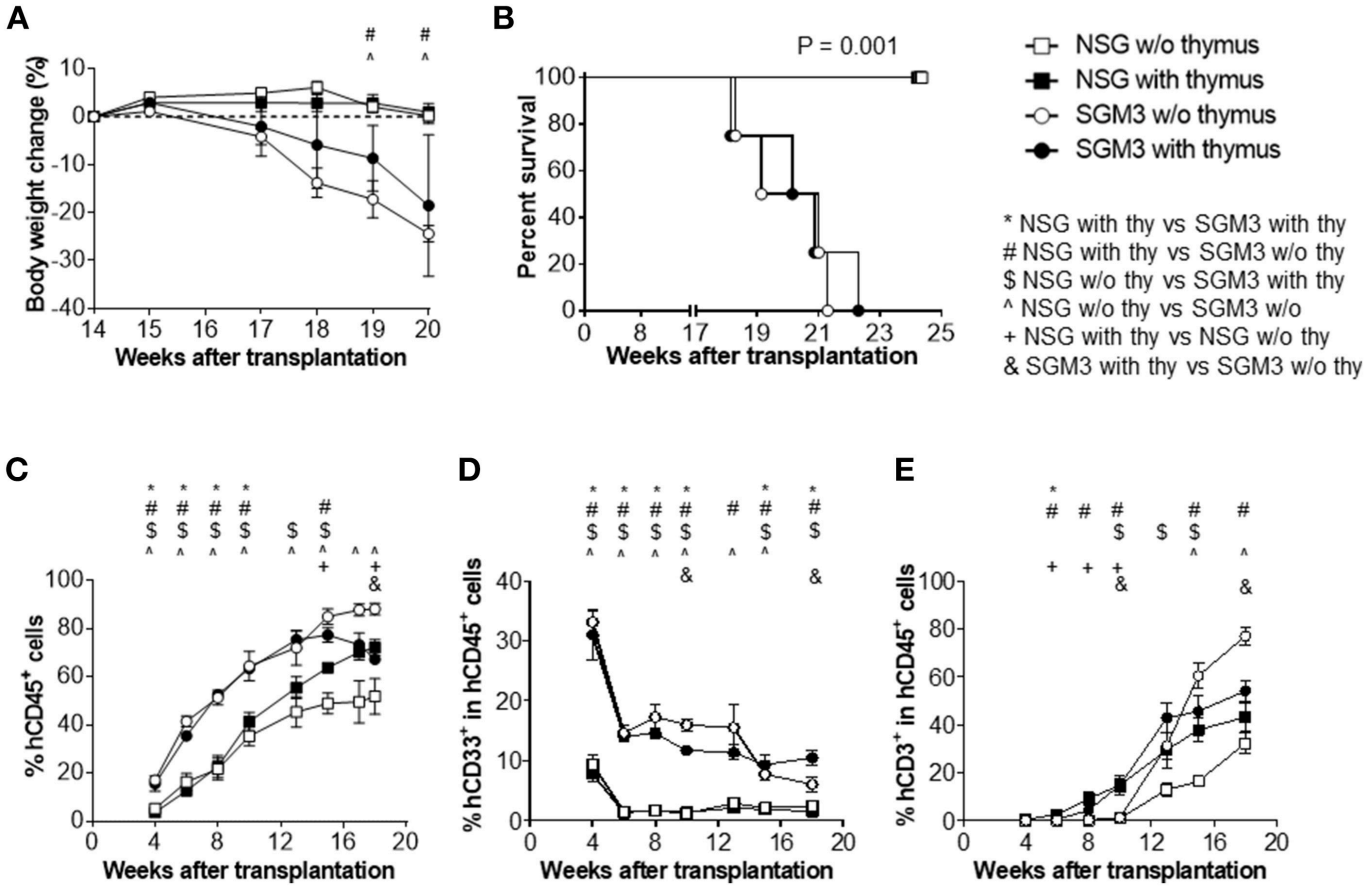
Body weight, survival and human blood cell reconstitution in huNSG and huSGM3 mice with or without human thymus. NSG or SGM3 mice were transplanted with human CD34^+^ cells alone or along with human fetal thymus tissue (*n* = 4 per group). **(A)** Body weight changes in the indicated groups of humanized mice between 14 and 20 weeks after transplantation. Body weight at 14 weeks was used as baseline value. **(B)** Survival of humanized mice after transplantation. **(C)** Levels (%) of human CD45^+^ cell chimerism in WBCs at the indicated time points after transplantation. **(D–E)** Kinetics of the frequencies of human CD33^+^ myeloid **(D)** and CD3^+^ T cells **(E)** within human CD45^+^ cells. For **(A,C–E)**, repeated measures analysis of variance was used to determine main effects (*P* < 0.05) between groups. All of the panels had main effects, and *post-hoc* Bonferroni was used to compare groups at each time point. For *p* < 0.05 for *post-hoc* test are indicated as *, #, $, or & for group comparisons indicated in the legend. Error bars represent SEMs.

Hu-mice were generated by intravenous injection of human CD34^+^ cells alone or along with implantation of human fetal thymus under the kidney capsule in sublethally irradiated NSG or SGM3 mice. All huSGM3 mice, regardless of with or without human thymus, became lethargic and started losing body weight from 17 weeks after transplantation (Figure 1A), and became moribund 18–22 weeks after transplantation (Figure 1B). With exception of one mouse with mild diarrhea, none showed signs of GVHD, such as ruffled fur or loss of skin integrity. In contrast to huSGM3 mice, huNSG mice, regardless of whether or not human thymus was transplanted, appeared healthy throughout the observation period (22 weeks), which is consistent with our previous studies (14).

### Higher Human Leukocyte Chimerism With Better Myeloid Reconstitution in HuSGM3 Mice

Peripheral blood was collected every 2–3 weeks starting from 4 weeks after transplantation and analyzed for human cell chimerism by flow cytometry. HuSGM3 mice (both with and without human thymus) showed higher human leukocyte (CD45^+^ cell) chimerism levels than huNSG mice (Figure 1C). Although the chimerism levels were similar between huSGM3 mice with and without human thymus until 13 weeks, those in the group without human thymus started to decline from 15 weeks after transplantation. Among huNSG mice, mice with human thymus had higher human leukocyte chimerism levels than those without throughout the observation period. Furthermore, huSGM3 mice had higher CD33^+^ myeloid cell frequencies than huNSG mice (Figure 1D). High myeloid cell frequencies at the early time (by 4 weeks) and subsequent decreases suggest that myeloid cells recovered first, followed by B cells and T cells as in the case of the patients undergoing hematopoietic stem cell transplantation. Human RBCs were almost undetectable in both huNSG and huSGM3 mice (data not shown), consisting with previous studies (14).

### Aggressive Peripheral T Cell Expansion in huSGM3 Mice, Particularly in the Absence of Human Thymus

It is known that human T cell reconstitution does not occur after CD34^+^ cells transplantation in the absence of human thymus graft when adult NOD/SCID mice are used to generate humanized mice (11). In fact, in both huNSG and huSGM3 mice, the no thymus group had scarce T cell reconstitution up to 10 weeks after transplantation (Figure 1E). However, T cell frequencies in these mice started to increase from 13 weeks, and the increase was strikingly steeper in huSGM3 than in huNSG mice. T cell frequencies in huSGM3 mice without human thymus even exceeded those in huSGM3 with human thymus by 15 weeks after transplantation. Interestingly, in groups with human thymus, the huSGM3 mice had only a slightly higher T cell frequency (not statistically significant) than the huNSG mice.

**Figure 2:**
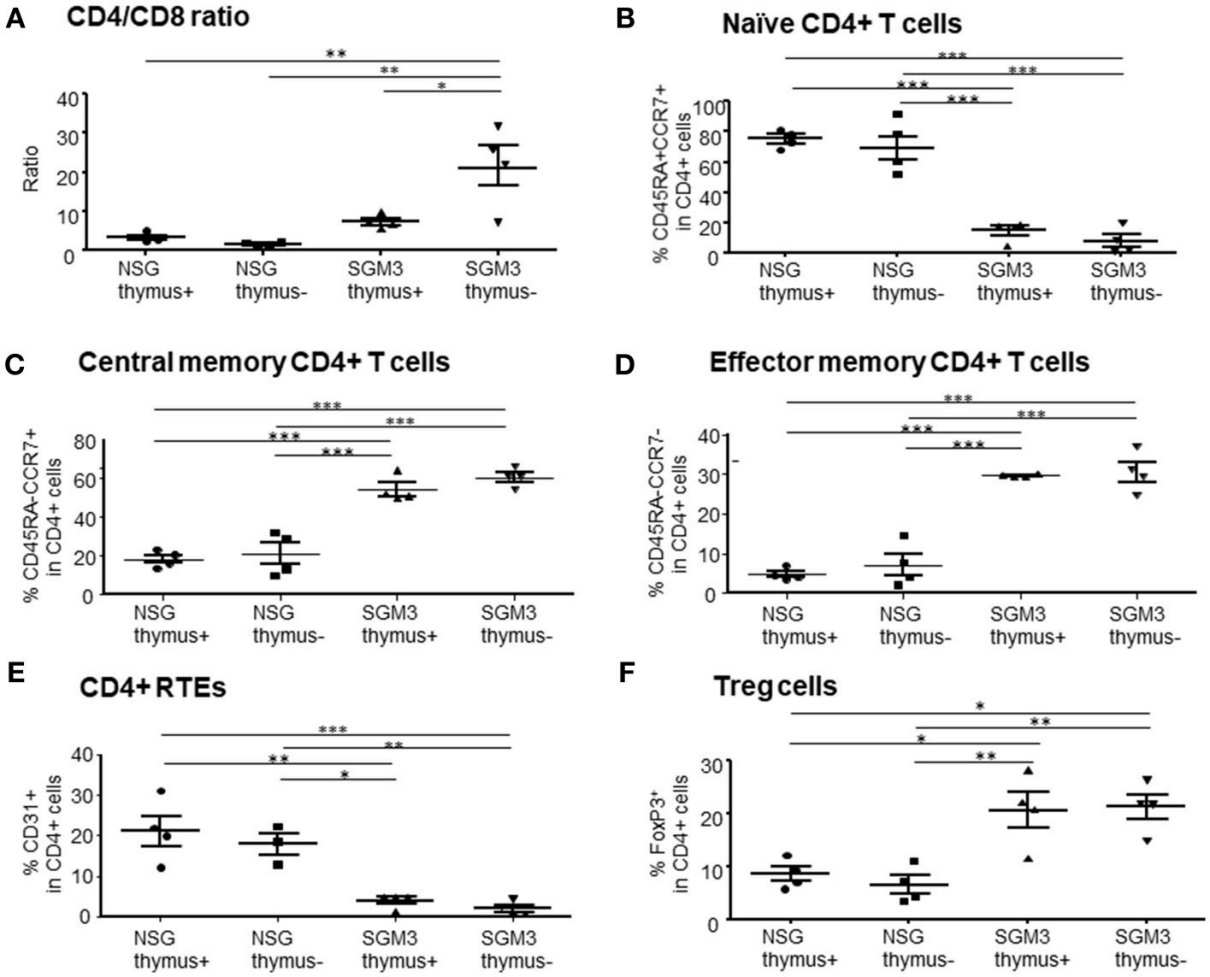
Characterization of human T cells in huNSG and huSGM3 mice with or without human thymus. Human T cells in peripheral blood at 15 weeks after transplantation were analyzed for the frequencies of T cell subsets (*n* = 4 per group). **(A)** Ratios of human CD4^+^ to CD8^+^ T cells within CD3^+^ population. **(B)** Percentages of human CD45RA^+^CCR7^+^ naïve CD4 T cells. **(C)** Percentages of human CD45RA^−^CCR7^+^ central memory CD4 T cells. **(D)** Percentages of human CD45RA^−^CCR7^−^ effector memory CD4 T cells. **(E)** Frequencies of CD31^+^CD45RA^+^ recent thymic emigrant CD4 T cells. **(F)** Frequencies of human FoxP3^+^CD25^hi^ Treg cells. Group differences were determined by one-way ANOVA with Bonferroni's *post-hoc* multiple comparison test; each symbol represents an individual mouse. *P < 0.05; **P < 0.01; ***P < 0.001. Error bars indicate SEMs.

We also performed detailed phenotypic analysis of T cells in peripheral blood at 15 weeks when the huSGM3 mice appeared to start losing weight. First, the CD4^+^ to CD8^+^ T cell ratio in huSGM3 mice was significantly higher than that in huNSG mice, in particular the huSGM3 mice without human thymus, in which more than 90% of CD3^+^ T cells were CD4^+^ T cells (Figure 2A). In huNSG mice (in both with and without human thymus groups), the majority of human CD4^+^ T cells expressed a naïve (CD45RA^+^CCR7^+^) phenotype (Figure 2B). In contrast, the majority of CD4^+^ T cells in huSGM3 mice expressed a central memory (CD45RA^−^CCR7^+^) or effector memory (CD45RA^−^CCR7^−^) phenotype (Figures 2C,D). Similar results were obtained from other experiments, in which augmented CD4^+^ T cell activation and expansion were detected in huSGM3 mice that were constructed by transplantation of bone marrow CD34^+^ cells and HLA-partially-matched fetal thymus compared to similarly constructed huNSG mice (Figure S1). CD31 has been described as a marker to identify the CD4^+^ recent thymic emigrants among the naive CD4^+^ T cell population (16). HuSGM3 mice also had significantly fewer CD31^+^ CD4^+^ T cells than huNSG mice (Figure 2E), likely due to abnormal human CD4^+^ T cell expansion and reduced human thymopoiesis (see below) in these mice. The results indicate an aggressive, human cytokine-driven expansion of human CD4^+^ T cells in huSGM3 mice, particularly in the absence of human thymus. The frequency of regulatory T cells (Tregs) defined as FoxP3^+^ cells within CD4^+^ T cells were higher in huSGM3 than in huNSG mice (Figure 2F), in concordance with a previous report (9).

### Severe Tissue Infiltration by Human T Cells and Hemophagocytic Macrophages in HuSGM3 Mice

**Figure 3:**
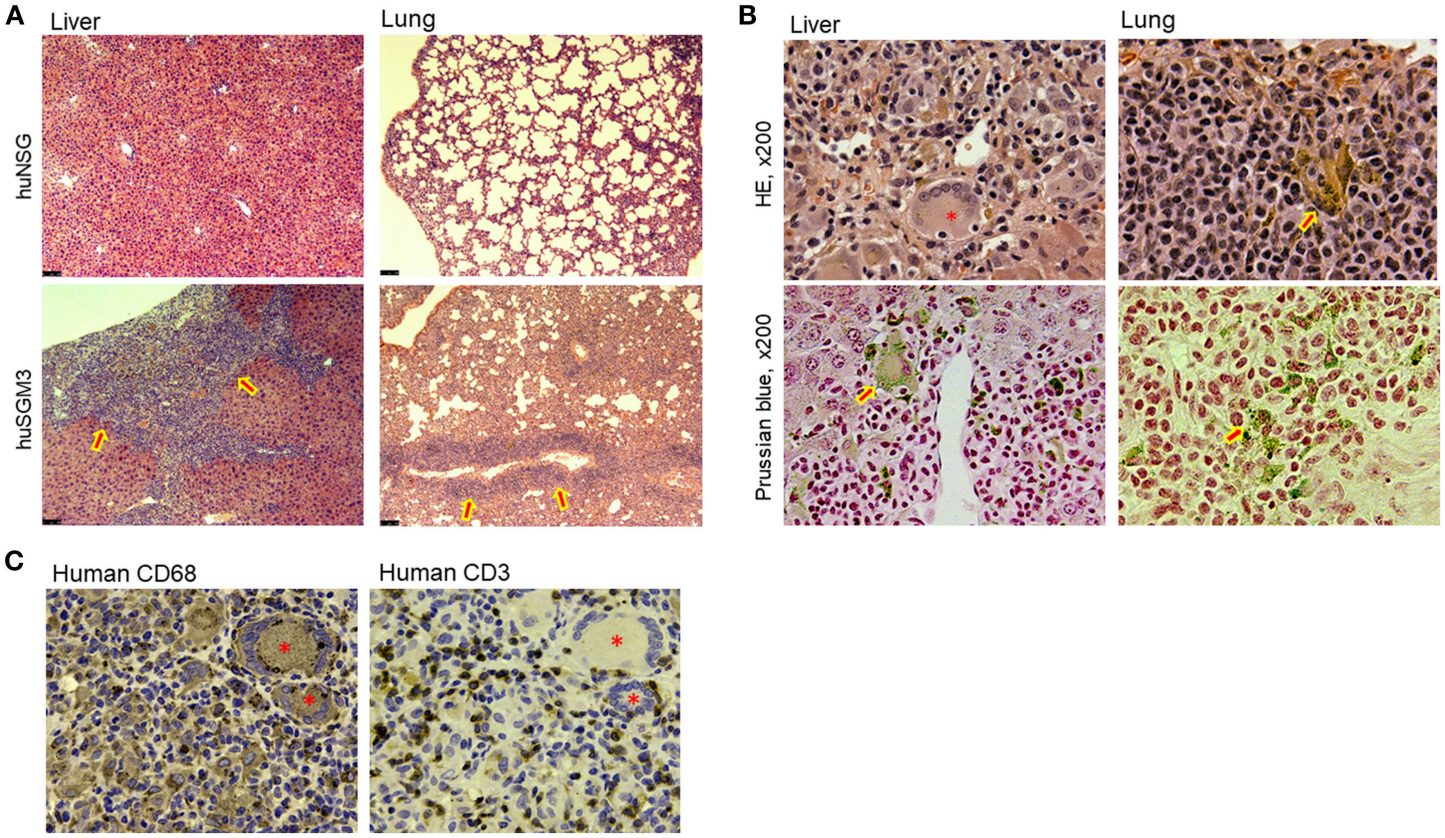
Tissue infiltration of T cells and hemophagocytic macrophages in huSGM3 mice. Liver and lung tissue samples were prepared from huNSG and huSGM3 mice 18–22 weeks after transplantation, and examined histologically (*n* = 4 per group). **(A)** Representative H&E staining of liver and lung tissue sections from huNSG and huSGM3 mice (without human thymus). Original magnification: x50. Arrow signs in huSGM3 mice organs show severe cellular infiltrations. **(B)** Representative staining images infiltration of hemosiderin-containing hemophagocytic histiocytes (macrophages) in liver and lungs from huSGM3 mice (without human thymus). Hemosiderin-containing hemophagocytic histiocytes, indicated by arrow signs, are stained with brown by H&E and blue by Prussian blue. *denotes a representative giant cell (i.e., fused macrophages) containing ingested erythrocytes. Original magnification: x200. **(C)** Representative immunohistochemical staining of huSGM3 mouse (without human thymus) liver sections (two consecutive sections) with anti-human CD3 and anti-human CD68 antibodies. *denotes a CD68^+^ giant cell.

To determine the cause of mortality in huSGM3 mice, comprehensive histopathological analysis was performed on various organs including liver, lungs, spleen, kidney, small intestine, colon, and skin. There were no significant findings in skin, small intestine, or colon (data not shown). However, liver from both of huSGM3 mice with or without thymus had strikingly severe cellular infiltration, which destructed normal structure (Figure 3A). Infiltrated cells consisted of lymphocytes and macrophages, and many of the latter are hemosiderin-containing hemophagocytic macrophages that were visualized brown by H&E staining and blue by Prussian blue staining (Figure 3B). In addition, giant cells, which are fused macrophages upon activation having multiple nucleus, were observed. Immunohistochemical staining with anti-human CD3 antibody and anti-human CD68 antibody confirmed that liver-infiltrating cells were predominantly human T cells and human macrophages, and distributed in a pattern with activated hemophagocytic macrophages being surrounded by T cells (Figure 3C). This pattern is different from that of GVHD in which T cells infiltrate predominantly into portal areas. Lungs also showed severe cellular infiltration that resulted in scarce alveolar space (Figure 3A), and the presence of large numbers of hemophagocytic macrophages that were stained positive with Prussian blue (Figure 3B). These observations indicate that the huSGM3 mice developed a fatal disease similar to HLH in human.

**Figure 4:**
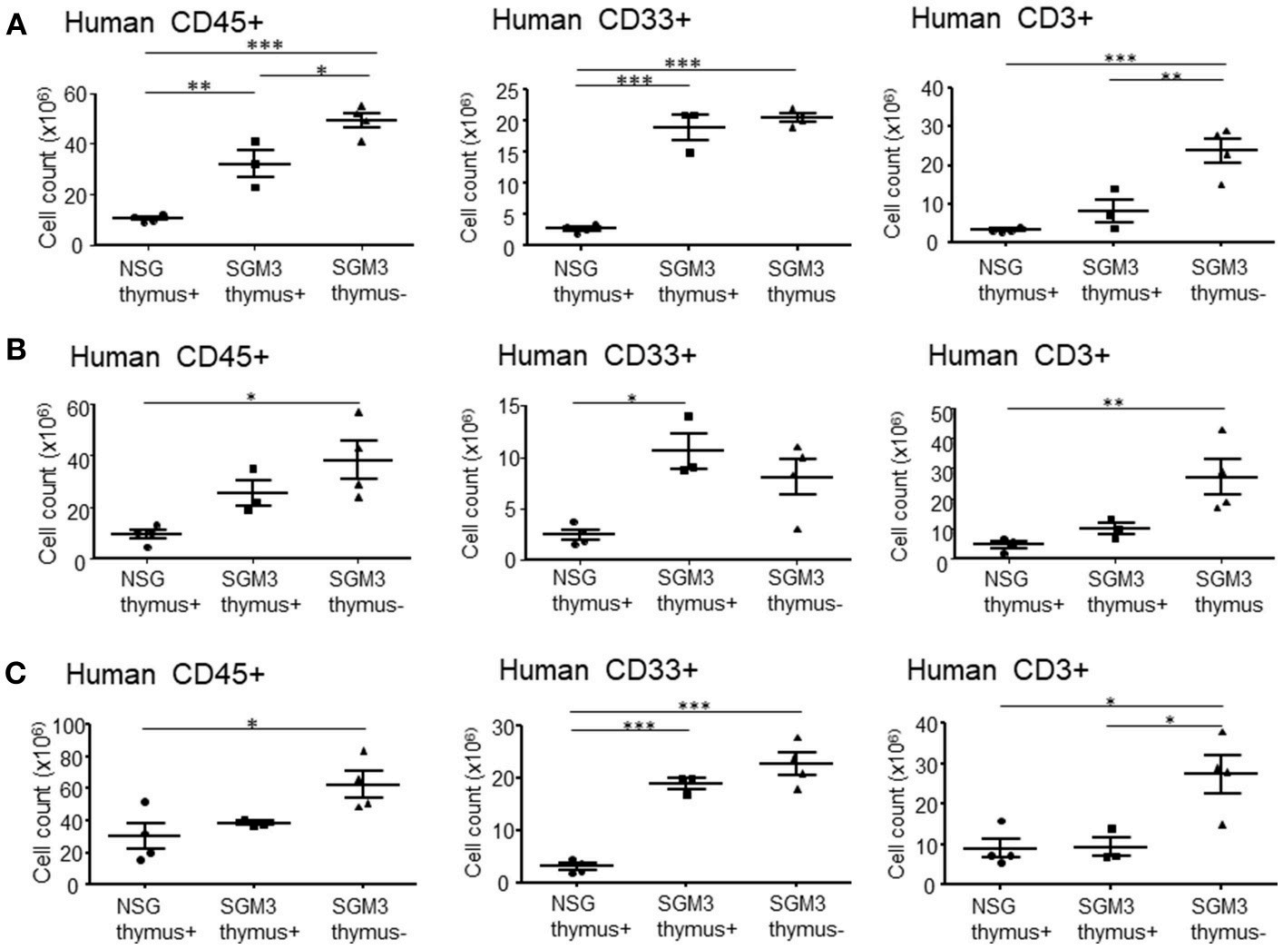
Absolute numbers of human T cells and myeloid cells isolated from huNSG and huSGM3 mice. HuSGM3 mice with or without human thymus graft (sacrificed when moribund between 18 and 22 weeks) and huNSG mice with human thymus graft (sacrificed at 21–22 weeks after transplantation) were analyzed for human cell infiltrations in various tissues (*n* = 4 per group). Absolute cell numbers of human CD45^+^ cells, T cells, and myeloid cells in liver **(A)**, lungs **(B)** and spleen **(C)** are calculated by multiplying the total cell count after mononuclear cell-purification using density gradient centrifugation with the percentage of each cell population determined by flow cytometry. One-way ANOVA with Bonferroni's *post-hoc* multiple comparison test; each symbol represents an individual mouse. *P < 0.05; **P < 0.01; ***P < 0.001. Error bars indicate SEMs.

We also performed flow cytometric analysis on single cell suspensions prepared from these tissues. In accordance with the histopathological analysis, absolute numbers of human CD45^+^ total leukocytes isolated from the liver of huSGM3 mice were significantly higher than those in huNSG mice (Figure 4A). Moreover, absolute numbers of human CD33^+^ myeloid cells isolated from the liver of huSGM3 mice were also significantly higher than those in huNSG mice, and were similar between with and without human thymus groups. Interestingly, huSGM3 mice without human thymus, but not those with human thymus showed significantly increased human T cell infiltration in the liver compared to huNSG mice. Lungs (Figure 4B) and spleen (Figure 4C) showed a similar pattern as the liver in absolute numbers of human CD45^+^ total leukocytes, CD33^+^ myeloid cells, and CD3^+^ T cells. These results indicate that human cytokines in the huSGM3 mice induced human myeloid and T cell infiltrations into the liver, lung and spleen, and suggested that the human thymus graft may suppress the infiltration of human T cells, but not myeloid cells, in these mice.

### Cytopenia in HuSGM3 Mice

**Figure 5:**
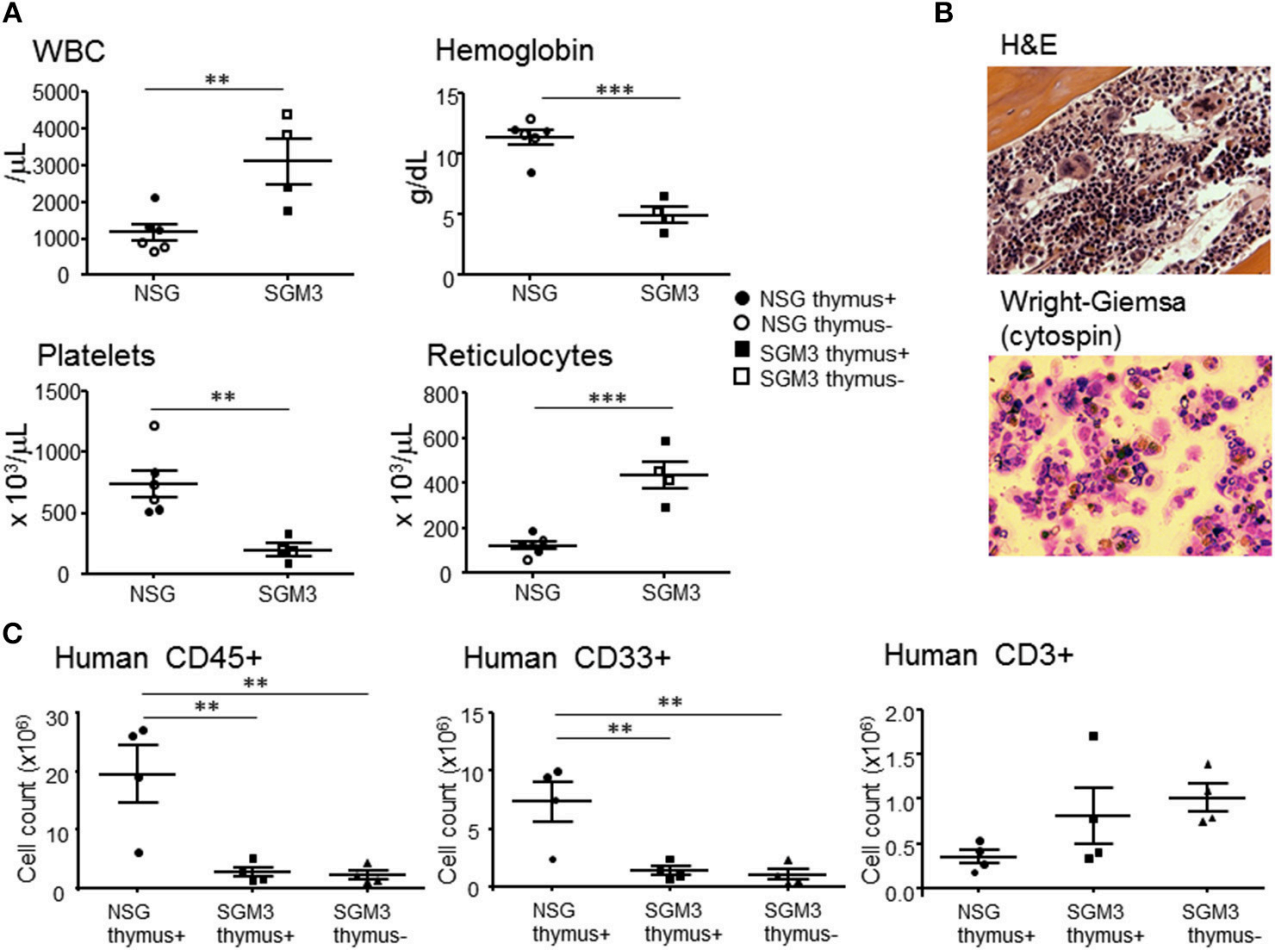
Cytopenia and bone marrow hypoplasia in huSGM3 mice. HuSGM3 mice with (closed square) or without (open square) human thymus were sacrificed when moribund between 18 and 22 weeks. HuNSG mice with (closed circle) or without (open circle) human thymus were sacrificed at 21–22 weeks after transplantation as controls. Since there was no apparent difference between the “thymus+” and “thymus-” groups, the data were pooled (but samples from different groups are distinguishable by their respective symbols). **(A)** White blood cell count (WBC), hemoglobin concentration, platelet count, and reticulocyte count measured by hematometry. Unpaired *t*-test; **P < 0.01; ***P < 0.001. Error bars indicate SEMs. **(B)** Histology of BM analyzed by H&E staining and cytology of BM analyzed by Wright-Giemsa staining. **(C)** Absolute cell numbers of human CD45^+^ cells, CD33^+^ myeloid cells and CD3^+^ T cells in BM, which were calculated by multiplying the total cell count after mononuclear cell-purification using density gradient centrifugation with the percentage of each cell population determined by flow cytometry. One-way ANOVA with Bonferroni's *post-hoc* multiple comparison test; each symbol represents an individual mouse. **P < 0.01.

Clinical symptoms of HLH in human include pancytopenia due to hypoplastic marrow caused by the myelosuppressive effects of inflammatory cytokines and hemophagocytosis (17). Despite a significantly increased white cell counts, moribund huSGM3 mice showed severe anemia and thrombocytopenia (Figure 5A). Interestingly, absolute reticulocyte counts in huSGM3 mice were significantly higher than that in huNSG mice (Figure 5A). Although hematopoietic stem cell exhaustion has been reported previously in the huSGM3 mice, (9, 18) the high reticulocyte counts in these mice suggest that destruction by activated macrophages, not poor production, is the major mechanism for anemia. In support of this possibility, histopathological analysis revealed hypoplastic marrow, with the presence of hemophagocytic macrophages and eosinophils in the huSGM3 mice (Figure 5B). Bone marrow hypoplasia was also confirmed by flow cytometry analysis showing a markedly reduced cellularity of human CD45^+^ and CD33^+^ myeloid cells in the bone marrow from huSGM3 mice (Figure 5C).

### Human Thymopoiesis in the Human Thymic Graft and Native Mouse Thymus in HuNSG and HuSGM3 Mice

**Figure 6:**
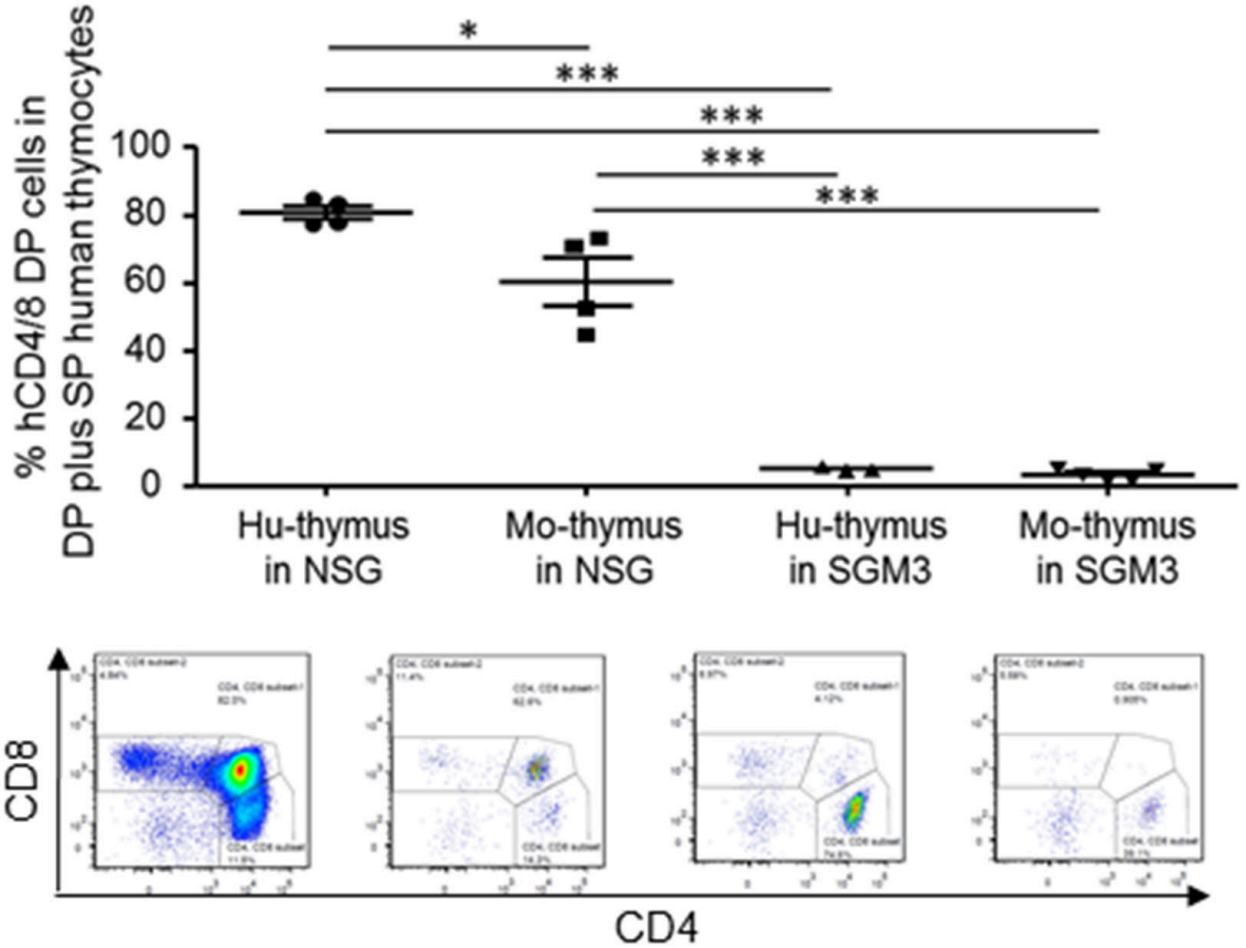
Thymopoiesis in the human and mouse thymus in NSG and SGM3 humanized mice. Frequencies (%) of CD4 and CD8 double positive cells (double positive cells / (double positive cells + single positive cells) × 100%) in the human and mouse thymus from huNSG and huSGM3 mice (with human thymic grafts) were shown with representative flow cytometric profiles. One-way ANOVA with Bonferroni's *post-hoc* multiple comparison test; each symbol represents an individual mouse. *P < 0.05; ***P < 0.001. Error bars indicate SEMs. DP, double positive, SP, single positive cells.

We also examined the function of human and mouse thymus in huNSG and huSGM3 mice. Briefly, we first identified human CD45^+^ cells, and then analyzed the expression of CD4 and CD8. Since some of the non-thymocytes might be contaminated in the CD4^−^CD8^−^ double negative (DN) population, we calculated the frequency of CD4^+^CD8^+^ double positive (DP) cells using DP cells plus CD4 and CD8 single positive (SP) cells as a denominator (Figure 6). In huNSG mice, human thymus graft had a higher DP cell frequency than mouse thymus. In contrast, in huSGM3, both human and mouse thymus had scarce DP cells, suggesting that thymopoiesis was severely damaged in these mice.

### Hypercytokinemia in HuSGM3 Mice

**Figure 7:**
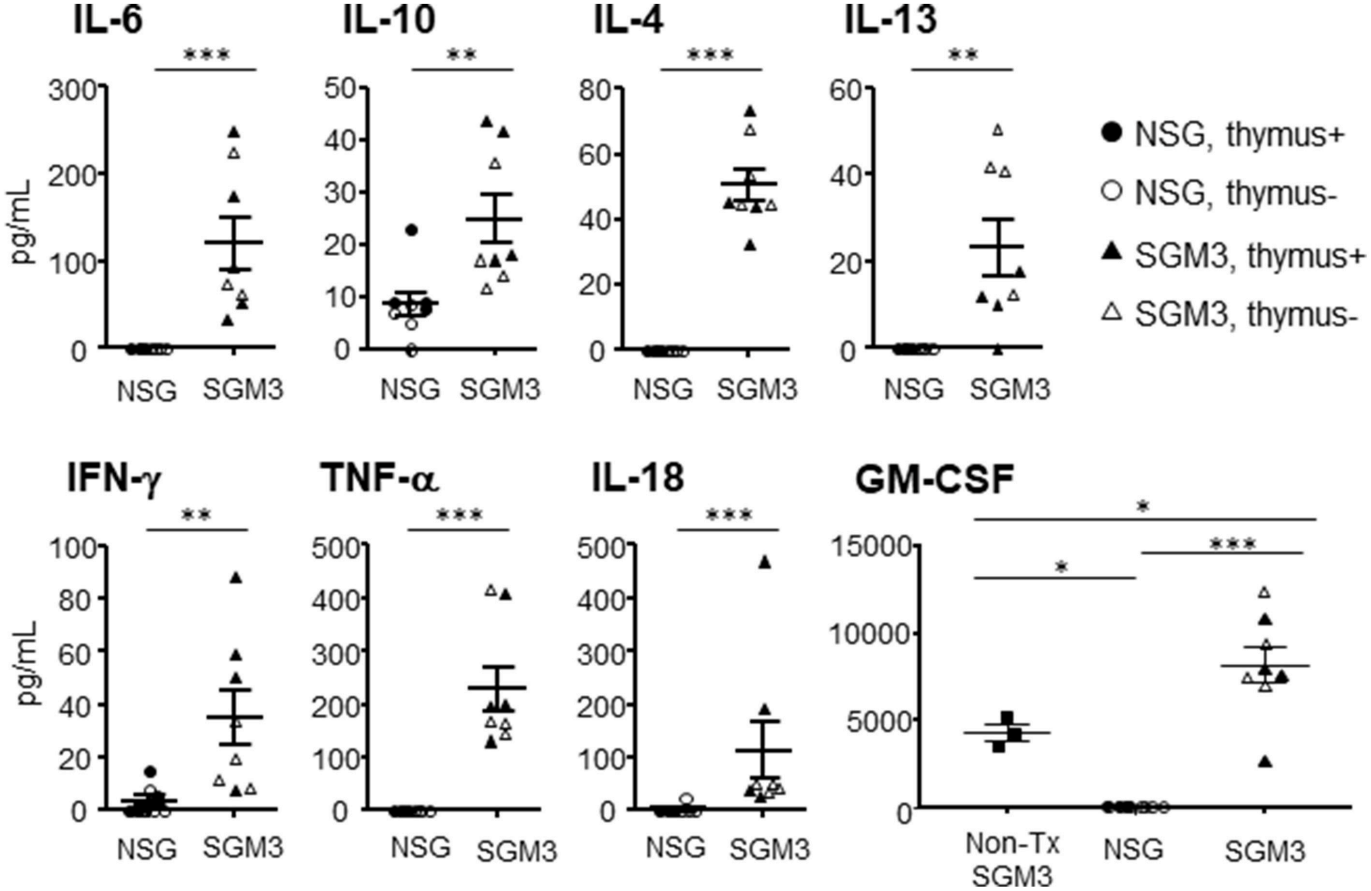
Plasma levels of human cytokines in huNSG and huSGM3 mice. Human cytokines levels in plasma at 18 weeks after transplantation were measured using multiplex immunoassay system. HuNSG and huSGM3 mice are shown as circle and square symbols, respectively, and each symbol represents an individual mouse (closed and open symbols represent mice with and without human thymus, respectively). Since there was no apparent difference between the “thymus+” and “thymus-” groups, the data were pooled (but samples from different groups are distinguishable by their respective symbols). Unpaired *t*-test; *P < 0.05; **P < 0.01; ***P < 0.001. Error bars indicate SEMs.

Plasma were prepared from huNSG and huSGM3 mice at 18 weeks after transplantation, and human cytokine levels were measured using the multiplex immunoassay system (Figure 7). The levels of human IL-4, IL-6, IL-10, IL-13, IL-18, IFN-γ, and TNF-α were significantly elevated in huSGM3 mice compared to huNSG mice. Plasma levels of IL-1β, IL-2, IL-5, and IL-12 were below sensitivity in both groups (data now shown). Interestingly, GM-CSF levels in huSGM3 mice were significantly higher than non-humanized SGM3 mice, reflecting the production of GM-CSF by activated human macrophages and/or T cells in the huSGM3 mice.

## Discussion

In the current study, we successfully reproduced a posttransplant HLH by utilizing huSGM3 mice and showed that posttransplant HLH is triggered by alloresponses (or xenoresponses in our model), driven by myeloid cytokines, and exacerbated by vicious cycles of T-cell and macrophage activation. HLH was seen in huSGM3 mice with or without human thymic grafts, indicating that mouse thymus-derived T cells were largely responsible for the disease development. Given that the huSGM3 mice with human thymus have a normal human immune T cell pool consisting of more naïve T cells and immune regulatory T cells (e.g., Tregs), these mice are considered more clinically relevant. However, because the disease is much more severe in huSGM3 mice without human thymus, these mice could also be helpful in testing the efficacy of HLH therapies. This study not only offers a humanized HLH mouse model, but also raises an alert on the use of human cytokine transgenic mice in the construction of hu-mice with functional human lymphohematopoietic systems in an attempt to improve myeloid reconstitution.

HLH is a life-threatening disease of severe hyper inflammation caused by uncontrolled proliferation of activated macrophages and lymphocytes that secrete high amounts of inflammatory cytokines, including IL-1, IL-6, IL-18, and TNF-α from macrophages and IFN-γ from T-cells. IL-10, an anti-inflammatory cytokine, is also elevated in HLH patients, (19) possibly as a response to hyper-inflammation (20). The cardinal symptoms include prolonged fever, hepatosplenomegaly, cytopenia, and hemophagocytosis by activated, morphologically benign macrophages. Recent studies have shown that hypercytokinemia is the driving cause of immunopathology of HLH, and most of above mentioned symptoms are attributable to hypercytokinemia. For example, fever is induced by IL-1 and IL-6, splenomegaly is the direct result of infiltration by lymphocytes and macrophages, and cytopenia can be explained by high concentrations of TNF-α and IFN-γ (21–23) as well as direct hemophagocytosis.

While HLH after allo-SCT has been recognized to be rare complication, recent studies have suggested that the incidence of posttransplant HLH is higher than previously thought, particularly in HLA-mismatched transplantation settings. Takagi reported that 11.8% of patients who received HLA-mismatched cord blood transplantation following reduced-intensity conditioning regimen developed HLH (3) Interestingly, they subsequently reported that the intensification of immune suppressant after SCT reduced the incidence of HLH (24). Jaiswal reported that 12.2% of patients who received HLA-haploidentical peripheral blood stem cell transplantation with posttransplant cyclophosphamide developed HLH (2). These findings support the theory that alloresponse is the trigger for the posttransplant HLH. Notably, huSGM3 mice in the current study closely resembled posttransplant HLH in the clinic. These mice manifested hepatosplenomegaly (Figure S2) with T cell and macrophage infiltrations and cytopenia, and had a significant elevation in multiple inflammatory cytokines including IL-6, IL-18, IFN-γ, and TNF-α. Compared to mouse models of primary HLH, which are generated by deletion or mutation of the murine orthologs of the genes involved in human primary HLH, mouse models of secondary HLH are scarce (25). Moreover, the majority of the limited number of secondary HLH models, including a model of EBV-associated HLH in hu-mice (26), are infection associated HLH/HPS models.

Although the current study did not specify which cytokine played a major role in the pathogenesis of HLH, GM-CSF is assumed to be the primary pathogenic cytokine, as the huSGM3 mice shared many of the findings observed in a mouse HLH model driven by GM-CSF overexpression, such as short survival, splenomegaly, lymphadenopathy, thymic atrophy, and multiple abnormalities in blood cell populations including progressing anemia (27). Nonetheless, such a humanized mouse model of HLH allows for examining the pathogenesis of secondary HLH caused by human immune systems, including the cross-talk between human T cells and macrophages via cytokines, and therefore, is more clinically relevant than previous mouse models and ideal for evaluating therapeutic options. Immunohistological analysis demonstrated severe tissue infiltration by activated human T cells and phagocytic macrophages, indicating that these human immune cells are the primary pathogenic effectors in HLH development in the huSGM3 mice. Moreover, since both human IL-6 and TNF-α, the major inflammatory cytokines driving the development of wasting syndrome or cachexia in HLH, are cross-reactive with mouse cells, (28) it is likely that the body weight loss and wasting syndrome are largely attributed to these human inflammatory cytokines. It has been reported that TNFα is capable of suppressing hematopoiesis by several mechanisms including direct cytotoxicity and induction of apoptosis (21–23). Therefore, elevated human TNFα production may also be one of the mechanisms causing severe hypocellularity in the BM in huSGM3 mice.

Another striking finding in our model was the delayed, but aggressive T cell reconstitution in huSGM3 mice without human thymus graft. We found that T cell frequencies in peripheral blood from both huNSG and huSGM3 mice without human thymus graft remained extremely low or almost absent until 10 weeks, but increased remarkably steeply thereafter in huSGM3 mice, and exceeded huSGM3 mice with human thymus by 15 weeks. Absolute T cell numbers from the organs proved that T cell expansion in the mice without human thymus graft was more aggressive than in the mice with human thymus.

These results suggest a possible aberrant human thymopoiesis in the mouse native thymus, leading to generation of functionally abnormal human T cells. Nevertheless, huSGM3 mice without human thymus did not show characteristic GVHD findings when they became moribund except for one mouse that had moderate diarrhea. These mice showed severe T cell infiltration of the liver; however, infiltration was not restricted to portal area as shown in GVHD, indicating it is more likely caused by uncontrolled proliferation due to hypercytokinemia. While it is plausible to assume that the huSGM3 mice developed lethal HLH prior to GVHD, another possible explanation could be the balance of myeloid and lymphoid cytokines determines the disease manifestations i.e., HLH or GVHD.

In conclusion, we successfully produced a disease model of posttransplant HLH. Our study suggests that posttransplant HLH is triggered by alloresponse (or xenoresponse in our model), driven by myeloid cytokines, and exacerbated by vicious cycles of T-cell and macrophage activation.

## Author Contributions

SY designed and performed experiments, analyzed data, and wrote the paper. YL, JX, and ND designed and performed experiments, and analyzed data. MS designed experiments and analyzed data. Y-GY conceived the research project, designed experiments, analyzed data, and wrote the paper. All authors edited and approved the manuscript.

### Conflict of Interest Statement

The authors declare that the research was conducted in the absence of any commercial or financial relationships that could be construed as a potential conflict of interest.
