# Supplementary material for: Posttransplant Hemophagocytic Lymphohistiocytosis Driven by Myeloid Cytokines and Vicious Cycles of T-Cell and Macrophage Activation in Humanized Mice
Source: Front Immunol. 2019 Feb 13;10:186. doi: 10.3389/fimmu.2019.00186 (PMC6381030; doi:10.3389/fimmu.2019.00186)
Supplement: Supplementary file 1 [file Data_Sheet_1.PDF]

Supplementary Figures

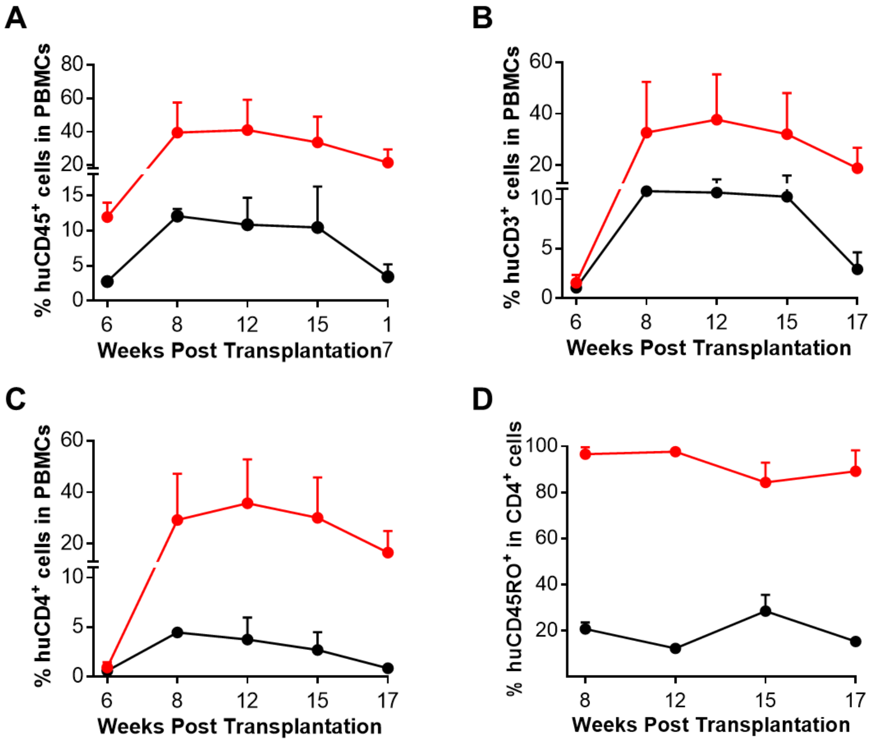

Figure S1. HuSGM3 mice show expansion of human T cells with an effector/memory phenotype compared to huNSG mice. Mice conditioned with 1.8Gy total body irradiation were injected with $2x10^5$ human bone marrow-derived CD34$^+$ cells and grafted with cryopreserved partially HLA-matched fetal thymus. PBMCs were enriched through ficoll gradient centrifugation and stained for flow cytometry starting 6 weeks post-transplantation. Average frequency of HuCD45$^+$ (A), CD3$^+$ (B), and CD4$^+$ (C) cells in PBMCs (mouse CD45$^+$ + human CD45$^+$ cells), and average frequency of CD45RA$^-$CD45RO$^+$ cells in human CD4$^+$ cells (D) are shown. Results representative of two independent experiments.

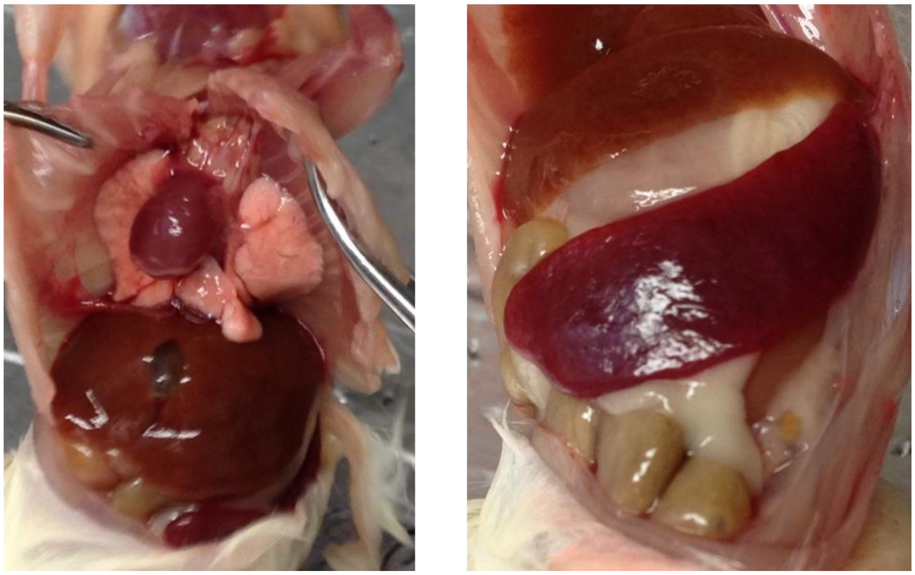

Figure S2. Macroscopic findings of huSGM3 mice with HLH. Autopsy was performed on huSGM3 mice that became moribund. Shown are macroscopic findings of a representative huSGM3 mouse (without thymus). (Left) A macroscopic picture showing pulmonary edema and hepatomegaly. (Right) A macroscopic picture showing severe splenomegaly.
